# Supplementary material for: Optical force mapping at the single-nanometre scale
Source: Nat Commun. 2021 Jun 23;12:3865. doi: 10.1038/s41467-021-24136-2 (PMC8222358; doi:10.1038/s41467-021-24136-2)
Supplement: Supplementary file 1 — Supplementary Information [file 41467_2021_24136_MOESM1_ESM.pdf]

# Supplementary Information

## Supplementary Note 1: Transmission electron microscopy images of ZAIS QDs

Transmission electron microscopy images of ZAIS QDs are shown in Supplementary Fig. 1, where a and b are the low- and high-magnification images, respectively. Supplementary Fig. 1b also represents the nanoscale structural features of the edge and rod, which are visualised in the PiFM image of Fig. 2b and c in the main text.

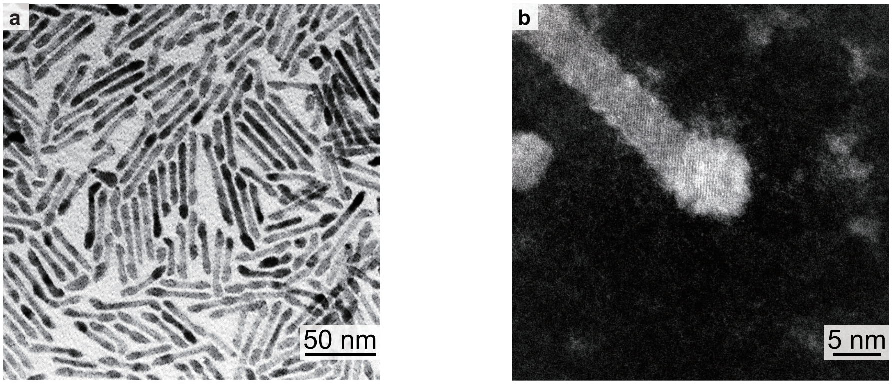


Supplementary Fig. 1: Transmission electron microscopy images of ZAIS QDs. **a**, Low-magnification TEM image. **b**, High-magnification HAADF-STEM image.

## Supplementary Note 2: Discussion of the gradient force and thermal expansion

It is important to evaluate whether the PiFM signal arises from the optical gradient force or the thermal expansion of the tip and sample.[1, 2] In the main text, we presented Fig. 1 and described how the PiFM signal ($\Delta f(f_{\mathrm{m}})$X) originates from the optical gradient force. The consistency of the experimental results with the theoretical calculations shows that the measured PiFM signal ($\Delta f(f_{\mathrm{m}})$X) does not correspond to the thermal expansion,[1, 2] associated with the light absorption, but to the gradient force directed toward the $z$ direction ($< F_{\mathrm{grad}} >_z \sim -3\alpha'_{\mathrm{t}}\alpha'_{\mathrm{s}}E_{0z}^2/2\pi z^4$), which is derived from the real part of the polarizability of the tip and sample ($\alpha'_{\mathrm{t,s}}$).[3, 4] Here, the equation for this gradient force is given under the dipole approximation, and $E_{0z}$ represents the incident electric field of $z$ direction. Under laser illumination at 660 or 785 nm, a large difference is evident between the nanoellipsoids and the nanorod, even though there is slight absorption at nanoellipsoid, as seen in Fig. 1c. This difference arises because it is the gradient force that is being measured. Unlike the absorption at 660 and 785 nm, the amount of light absorbed at 520 nm greatly differs between the nanoellipsoids and nanorod. However, such a large difference in the photoinduced force is not observed in Fig. 1f. This observation implies

that the detected signal does not reflect the thermal expansion of the sample as it does in AFM-based infrared spectroscopy (AFM-IR).[5]

Here, we consider the issue of thermal expansion from some additional perspectives. First, we simulated the thermal expansion of a QD on a gold surface using COMSOL Multiphysics 5.3a. In the simulation model, a gold-coated Si tip with a radius of 30 nm was placed on a gold substrate with a thickness of 100 nm. The distance between the tip apex and the gold surface was 10 nm. The thickness of the gold coating on the tip was 40 nm. Spherical crystals of InAs were arranged as QDs on the gold substrate. The radius of each QD was 4 nm. The contact surface between each QD and the gold substrate was a circle with a radius of 800 pm. Here, the incident laser light had a wavelength of $\lambda = 660$ nm and an incidence angle of 70 °. The intensity of the laser irradiation was $P = 1.0 \times \sin(2\pi 100\text{k}t)$ kW in an area with a 20 $\mu$m radius from the QD. As the simulation geometry was two-dimensional, the system was assumed to be axially symmetric. The calculated heat generation mapping is shown in Supplementary Fig. 2a. Heat is strongly generated on the QD. Supplementary Fig. 2b shows the temperature mapping when the temperature of the QD reaches its maximum in the 100$^{\text{th}}$ cycle. The temperature at the QD is almost the same as that of the substrate despite the strong heat generation in the QD. Supplementary Fig. 2c plots the temporal temperature variations from the 90$^{\text{th}}$ to 100$^{\text{th}}$ cycle at the c point and at a point 20 $\mu$m from the c point (the $\sigma$ point). As shown in Supplementary Fig. 2c, the temperature variations at the c and $\sigma$ points are almost identical. Thus, it can be understood that the heat generated on the QD and the tip minimally influences the temperature of the sample. Furthermore, to confirm the effect of heat generation in the QD, the heat generation mapping without the QD and the corresponding mapping at the maximum temperature are shown in Supplementary Figs. 2d and e. As shown in Supplementary Fig. 2d, the heat generation values are smaller in the absence of the QD. However, the sample temperature in Supplementary Fig. 2e is almost the same as that in Supplementary Fig. 2b. Supplementary Fig. 2f shows the temporal temperature variations on the thin gold film at the c point with and without the QD. These temperature variations almost entirely overlap. From these results, it is found that the temperature at the QD depends only on the temperature variations of the gold substrate, not on the heat generated by the QD.

Next, because we only needed to investigate the substrate temperature, as discussed above, we simulated the temperature variations of the gold substrate under laser irradiation. The laser was focused on the gold substrate, and the laser was assumed to generate heat with a Gaussian distribution (Supplementary Fig. 3a). Here, the generated heat is expressed as $Q = G(x) \times \sin(2\pi 100\text{k}t)$ J, where $G(x)$ is a normal distribution with a standard deviation of $\sigma = 20$ $\mu$m. The DC component of the heat generation was not considered because it does not affect the modulation measurement. The thickness of the gold substrate ($d$) was 100 nm. The temperature variations from the 90$^{\text{th}}$ to 100$^{\text{th}}$ cycle are plotted in Supplementary Fig. 3b. As seen from this figure, a phase delay of $\sim$ 90 °is evident both at the center of the focused light spot and at a point $\sigma/2$ from the center. From these results, it is found that a temperature change near the laser spot with an $\sim$ 90 °phase delay appears. Under the assumption that the light is aligned with the tip with an accuracy of approximately $\sigma/2$ during the measurement, the phase delay is $\sim$ 97.2 °. Considering the phase delay calculated in this simulation, the influence of the thermal expansion of $\sim$ 87.5 % can be avoided by observing the nondelayed PiFM signal ($\Delta f(f_{\text{m}})$X).

As our next step, we describe the origin of the phase delay due to thermal expansion. This phase delay is explained by the thermal expansion, which is shown as follows.

$$\rho C \frac{dT}{dt} = Q(\boldsymbol{r}, t) - \kappa_{\text{eff}} \nabla^2 T. \tag{1}$$

Here, $T$ and $Q$ are the temperature and the heat generated in the sample, respectively, at site ($\boldsymbol{r}$), and $\rho$, $C$, and $\kappa_{\text{eff}}$ are the density, specific heat capacity at a constant volume, and thermal diffusion coefficient, respectively, of the sample. When the temperature change accompanying the heat generation due to the intensity modulation of the laser is sufficiently larger than the temperature change due to thermal diffusion, the modulation frequency

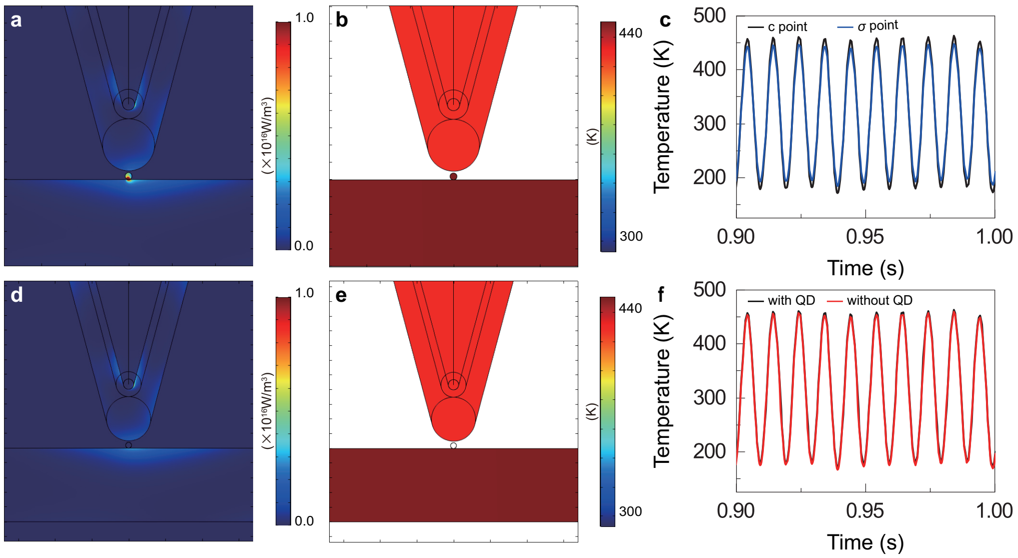


Supplementary Fig. 2: Simulation results for the laser-irradiated tip and sample. **a**, **d**, Heat generation mappings with and without a QD. **b**, **e**, Maximum-temperature mappings with and without a QD. **c**, Temporal temperature variations from the 90$^{\text{th}}$ to 100$^{\text{th}}$ cycle at the c and $\sigma$ points with a QD. **f**, Temporal temperature variations from the 90$^{\text{th}}$ to 100$^{\text{th}}$ cycle at the c point with and without a QD.

is sufficiently high, and the thermal diffusion equation can be approximated as follows:

$$\rho C \frac{dT}{dt} = Q(\boldsymbol{r}, t). \tag{2}$$

In a periodic heating state, the temporal temperature variation of the sample is expressed as $T = \Delta T \exp(i\omega t - \pi/2)$. Here, $\Delta T$ is the amplitude of the deviation of the temperature, and $\omega$ is the angular frequency of the laser power modulation. The phase is delayed by 90 °. Therefore, the thermal expansion of the sample is also delayed by $\sim$ 90 °with respect to the intensity modulation of the light. Next, the theoretical expression for the gradient force on the tip under the dipole approximation is shown below.[3, 4]

$$< \boldsymbol{F} >= \frac{\alpha'}{4} \nabla < |\boldsymbol{E}|^2 > + \omega_{\text{light}} \alpha'' < \boldsymbol{E} \times \boldsymbol{B} >, \tag{3}$$

where $\alpha'$ and $\alpha''$ are real and imaginary parts of the tip, and $\omega_{\text{light}}$ is the angular frequency of the incident light. As seen from this equation, there is no phase delay of the photoinduced force accompanying the laser intensity modulation. From these facts, it can be understood why the PiFM signal reflecting the thermal expansion exhibits a 90 °phase delay with respect to the dipole–dipole interaction signal.

This phase delay was also experimentally observed in the PiFM measurements. Here, we illustrate the behaviour of the tip–sample thermal expansion as observed by directly measuring the PiFM signal. To observe the thermal expansion, the tip was brought into the contact region on a nanoellipsoid at the end of a ZAIS QD. The acquired force curves of the photoinduced force are shown in Supplementary Fig. 4a; to obtain each curve plotted in this figure, four force curves were averaged to achieve a better signal-to-noise ratio. When the distance between the tip and the sample was slightly different from 0 nm, only the LIX signal was obtained. In contrast, the LIX signal abruptly decreased and the LIY signal suddenly increased when the tip–sample distance

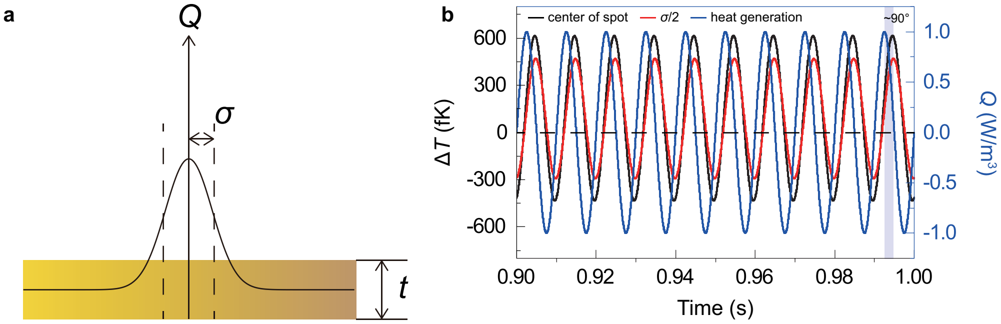


Supplementary Fig. 3: Substrate heating simulation. **a**, Model of substrate heating. Here, the heat generation mapping has a standard Gaussian distribution with $\sigma = 20\ \mu$m and $d = 100$ nm. **b**, Temperature variations from the 90$^{\text{th}}$ to 100$^{\text{th}}$ cycle at the c and $\sigma/2$ points. The heat generation profile is also shown.

became equal to $\sim$ 0 nm. This drastic change in the signals is similar to that for a PiFM signal detecting thermal expansion in the contact region.[1] This drastic signal change produces a drastic change in the phase of the PiFM signal, as shown in Supplementary Fig. 4b. At positions where the probe is far from the sample surface, only the LIX signal gradually increases, as indicated by the blue arrow. Then, a sudden phase change, as indicated by the red arrow, is detected in the contact region. This phase change is equal to approximately 90 °. Considering these results, it seems that the decrease in the LIX signal in the contact region is due to weakening of the plasmon enhancement or to tunnelling of electrons from the tip to the sample, resulting in a decrease in the dipole-dipole interaction.[6] In addition, the large LIY signal, which represents the force detected in the contract region, reflects the thermal expansion of the sample. Consequently, the 90 °phase shift is reasonable.[1]

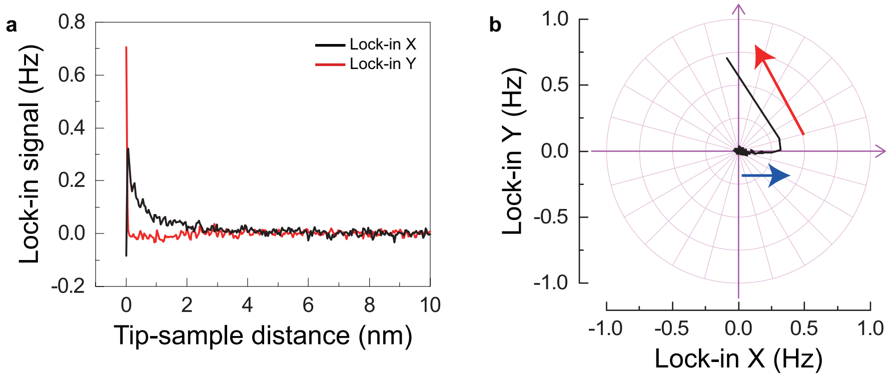


Supplementary Fig. 4: **a**, Force curves at the end of a ZAIS QD. The tip is in contact with the QD at $z \sim 0$ nm. **b**, Phase change in the force curves visualized in the LIX–LIY plane.

Moreover, images of a ZAIS cluster on the thin gold film show the same phase shift behaviour. Supplementary Figs. 5a and b show AFM and PiFM phase images, respectively. During the imaging process, the probe was in contact with the end of the ZAIS QD. In the PiFM phase image (Supplementary Fig. 5b), it can be seen that the phase of the PiFM signal, not $\Delta f(f_{\mathrm{m}})$X but $\Delta f(f_{\mathrm{m}})$, markedly changes. As seen from the line profile in c, the phase change was $\sim$ 82 °compared with the PiFM signal on the gold thin film. Therefore, the contribution of the thermal expansion appears in the PiFM signal with a phase deviation of $\sim$ 90 °even in imaging.

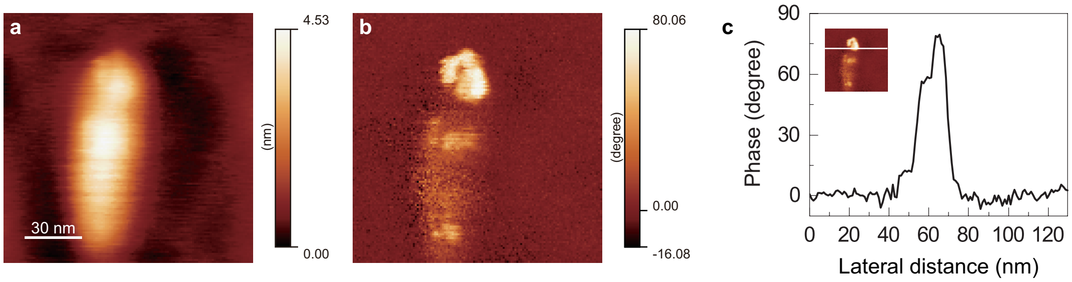


Supplementary Fig. 5: ZAIS cluster imaging. **a**, AFM image. **b**, PiFM phase image. **c**, Line profile of the image shown in **b**.

Supplementary Figs. 6a, b, c, and d show LIX and LIY images of the cluster shown in Supplementary Fig. 5b obtained via PiFM. The laser wavelengths were $\lambda = 660$ nm (Supplementary Figs. 6a and b) and $\lambda = 785$ nm (Supplementary Figs. 6c and d). Regardless of the wavelength, the PiFM LIX images (Supplementary Figs. 6a and c) show a reduction in the signal at the point where the probe is in contact with the sample. By contrast, the PiFM LIY images (Supplementary Figs. 6b and d) show an increase in the signal at the contact site. Moreover, the behaviours observed in the LIX and LIY images are the same as those observed from the force curves (Supplementary Fig. 4a), i.e., a decrease in the LIX signal and an increase in the LIY in the contact region. In addition, although the QDs show no absorption at 785 nm, thermal expansion is detected in the corresponding PiFM image. This result experimentally supports the simulation results indicating that the temperature variation of the substrate, rather than the heat generation in the QDs, dominantly influences the temperature of the QDs.

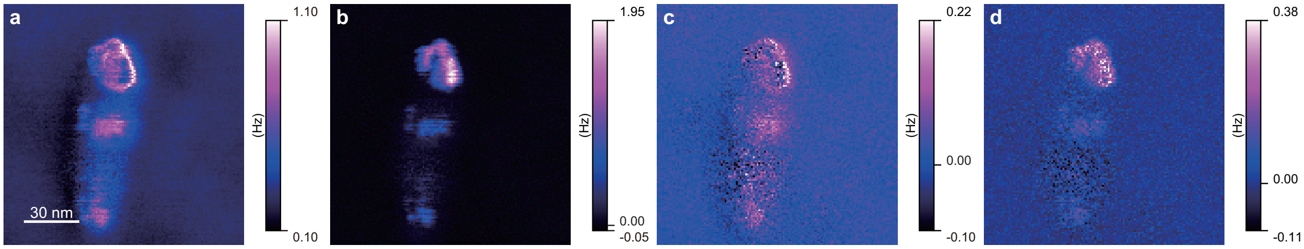


Supplementary Fig. 6: ZAIS cluster PiFM LIX and LIY images. **a**, **b**, LIX and LIY images at $\lambda = 660$ nm. **c**, **d**, LIX and LIY images at $\lambda = 785$ nm.

Up to this point, we have discussed the thermal expansion based on the differences in the phases of the detected signals. Hereafter, we discuss the thermal expansion from a different perspective, i.e., the shape of the force curves. By acquiring PiFM force curves and comparing them, we can determine whether the force detected is the gradient force or the thermal expansion. If the thermal expansion of the tip and sample is detected, then all forces except for the photoinduced force will be modulated by the thermal expansion.[2] In this case, the PiFM force curve reflects the shape (order) of the force curve of the non-photoinduced forces. In other words, when the non-photoinduced forces are the same, the PiFM force curves should be of the same order. We compare the force curves measured on the nanoellipsoid and the nanorod of a ZAIS QD (Supplementary Fig. 7a, $\Delta f(f_{\mathrm{m}})\mathrm{X} - z$; Supplementary Fig. 7b, $\Delta f - z$). Here, to reveal the differences in the photoinduced forces in the images, the force curves were acquired with a feedback control ($\Delta f = -28$ Hz). The PiFM force curves (Supplementary Fig. 7a) show different attenuation lengths on the nanoellipsoid and nanorod of the QD, although the frequency

shift force curves (Supplementary Fig. 7b) at the two sites are quite similar. Each of the $\Delta f(f_\mathrm{m})$X curves were fitted to the function $a/(z+b)^\mathrm{n}$, and the evaluated parameters are $a = 0.71$ Hz, $b = 0.86$ nm, and $n = 2.4$ on the nanoellipsoid and $a = 0.22$ Hz, $b = 0.70$ nm, and $n = 1.45$ on the nanorod. The large difference in $n$ indicates that the detected PiFM signal does not reflect the thermal expansion. As another conceivable thermal effect, a force could also be generated by a water layer on the sample, which could enhance the thermal expansion signal. However, no such force can exist in our measurements because of the UHV observation conditions. Therefore, we conclude that the detected PiFM signal reflects the optical gradient force.

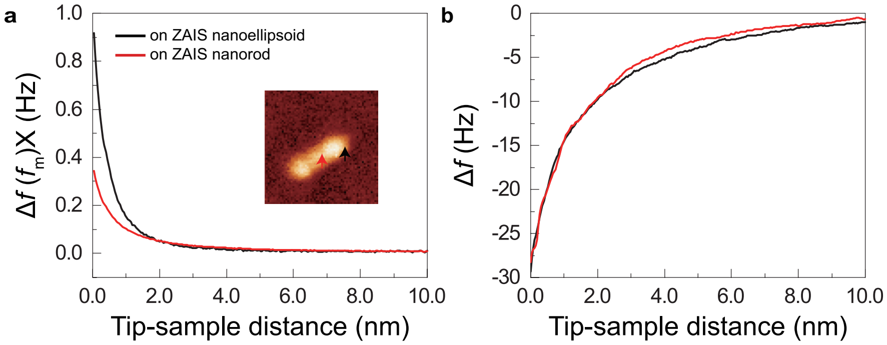


Supplementary Fig. 7: **a**, PiFM LIX force curves on a ZAIS nanoellipsoid and nanorod. **b**, Resonance frequency shift force curves measured via FM-AFM.

## Supplementary Note 3: Feedback error of the high-resolution imaging

Here, we consider the feedback error in the imaging of Fig. 2 from the main text to exclude the possibility that the tiny structure observed in the PiFM images is originated by the feedback error of the distance control between the tip and sample. We show the AFM image and feedback signal image ($\Delta f$) in Supplementary Fig. 8a and b, respectively. The feedback signal is defined as $-20$ Hz. The difference of $\Delta f$ from $-20$ Hz is the feedback error. We can estimate the feedback error in our experiment as about $\pm 0.02$ Hz. Apparently, we understand that the feedback error is almost negligible, and we do not worry about the effect of the cross-talk.

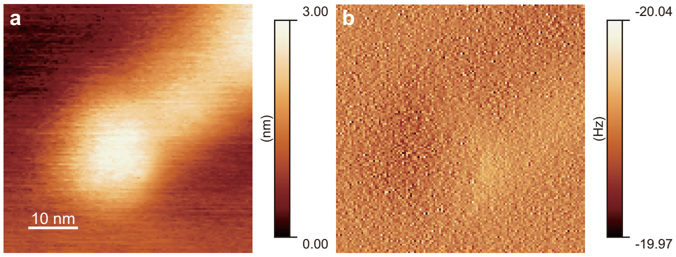


Supplementary Fig. 8: **a**, Topographic image ($\Delta z$) of the edge of the ZAIS QD. **b**, Feedback signal image ($\Delta f = -20$ Hz). The difference of $\Delta f$ from $-20$ Hz is the feedback error.

## Supplementary Note 4: High-resolution imaging

High-resolution images were obtained even when we used other tips and QDs as shown in Supplementary Fig. 9.

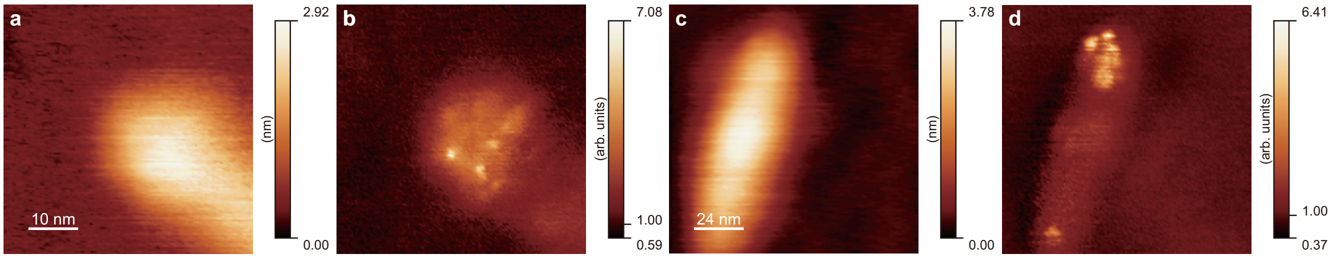


Supplementary Fig. 9: High-resolution ZAIS QD imaging. **a**, **b**, AFM and PiFM images of a ZAIS QD edge. **c**, **d**, Other AFM and PiFM images of a ZAIS QD. $\lambda = 660$ nm for both of the PiFM images.

## Supplementary Note 5: Exploratory factor analysis

In this paper, noise reduction based on exploratory factor analysis (EFA) was performed in the three-dimensional mapping. Here, to demonstrate that the noise could be successfully eliminated, comparisons of the spectra before and after the noise reduction are shown in Supplementary Fig. 10.

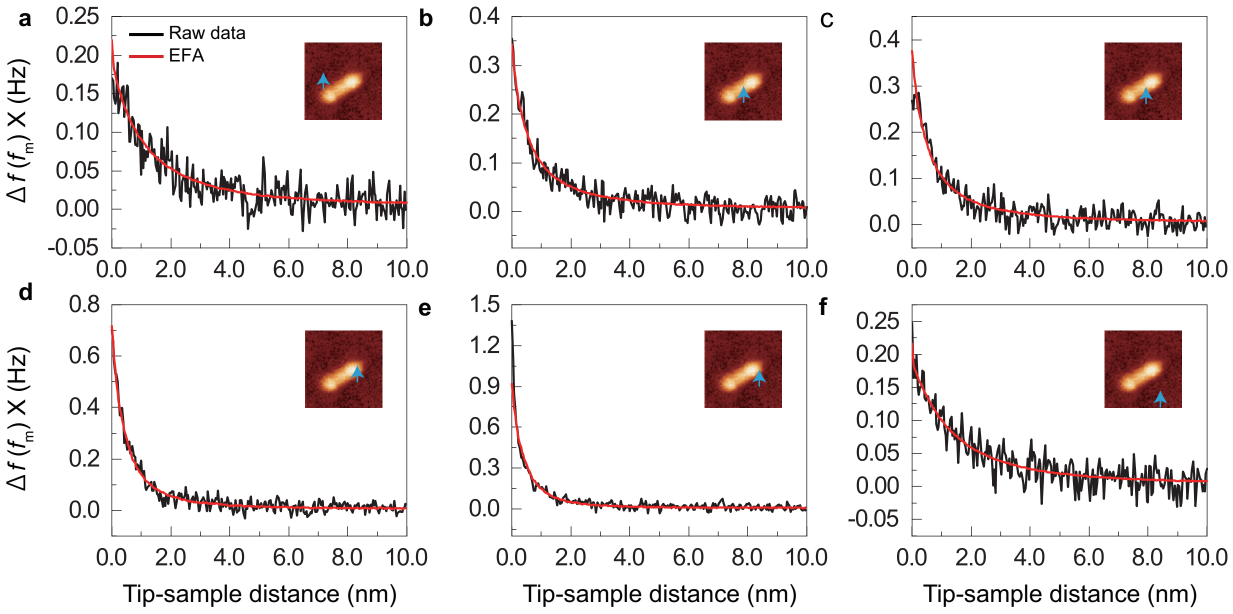


Supplementary Fig. 10: $\Delta f(f_\mathrm{m})\mathrm{X} - z$ spectra taken from raw and EFA-processed data. The arrows in the insets indicate the positions where the spectra were acquired.

## Supplementary Note 6: Frequency shift-to-force conversion in the heterodyne-FM technique

The expression for the calculation of the photoinduced force ($F_{\rm pif}$) from $\Delta f(f_{\rm m})$X in the heterodyne-FM technique is discussed in the following. Here, we introduce the calculation procedure for calculating $F_{\rm pif}$ in the heterodyne-FM technique at an arbitrary amplitude. The shift in the resonance frequency of the cantilever in FM-AFM is expressed as shown below.[7]

$$\Delta f = -\frac{f_0}{2ka_1}\int_{\phi}^{2\pi/\omega+\phi} F_{\rm ts}(z)\cos(\omega t+\phi_1)dt. \tag{4}$$

Here, $f_0$, $k$, and $a_1$ are the first resonance frequency without any perturbation, the spring constant, and the amplitude of the cantilever, respectively. $\phi$ is the arbitrary phase for this integration, and $\phi_1$ is the phase of the oscillation of the cantilever. Considering that the intensity of the laser beam is modulated, the force between the tip and sample becomes $F_{\rm ts}(z) = F_{\rm nonpif}(z) + F_{\rm pif}(z)\Big[1+\cos\{(2\omega+\omega_{\rm m})t+\phi_{\rm m}\}\Big]$. Here, $F_{\rm nonpif}$ is the non-photoinduced force, $\omega_{\rm m} = 2\pi f_{\rm m}$, and $\phi_{\rm m}$ is the phase of the modulation. Thus, Eq. (4) takes a new form as follows:

$$\begin{aligned}\Delta f = &-\frac{f_0}{2ka_1}\int_{\phi}^{2\pi/\omega+\phi} \{F_{\rm nonpif}(z)+F_{\rm pif}(z)\}\cos(\omega t+\phi_1)dt\\ &-\frac{f_0}{2ka_1}\int_{\phi}^{2\pi/\omega+\phi} F_{\rm pif}(z)\cos\{(2\omega+\omega_{\rm m})t+\phi_{\rm m}\}\cos(\omega t+\phi_1)dt\\ &=\Delta f(0)-\frac{f_0}{2ka_1}\int_{\phi}^{2\pi/\omega+\phi} F_{\rm pif}(z)\cos\{(2\omega+\omega_{\rm m})t+\phi_{\rm m}\}\cos(\omega t+\phi_1)dt.\end{aligned} \tag{5}$$

For the second term,

$$\begin{aligned}&-\frac{f_0}{2ka_1}\int_{\phi}^{2\pi/\omega+\phi} F_{\rm pif}(z)\cos\{(2\omega+\omega_{\rm m})t+\phi_{\rm m}\}\cos(\omega t+\phi_1)dt\\ &=-\frac{f_0}{2ka_1}\int_{\phi}^{2\pi/\omega+\phi} F_{\rm pif}(z)\frac{1}{2}\Big[\cos\{(3\omega+\omega_{\rm m})t+\phi_{\rm m}+\phi_1\}+\cos\{(\omega+\omega_{\rm m})t+\phi_{\rm m}-\phi_1\}\Big]dt\\ &=-\frac{f_0}{4ka_1}\int_{\phi}^{2\pi/\omega+\phi} F_{\rm pif}(z)\{\cos(3\omega t+\phi_1)\cos(\omega_{\rm m}t+\phi_{\rm m})\\ &\quad-\sin(3\omega t+\phi_1)\sin(\omega_{\rm m}t+\phi_{\rm m})\\ &\quad+\cos(\omega t-\phi_1)\cos(\omega_{\rm m}t+\phi_{\rm m})\\ &\quad-\sin(\omega t-\phi_1)\sin(\omega_{\rm m}t+\phi_{\rm m})\}dt.\end{aligned} \tag{6}$$

Considering that $\omega \gg \omega_{\rm m}$, the cosine function with the angular frequency of $\omega_{\rm m}$ in the integral can be considered to take an approximately constant value, this function can be moved outside of the integral. Furthermore, given that the component with the angular frequency of $3\omega$ in $F_{\rm pif}(z)$ is sufficiently small, the first and second terms in the integral in Eq. 6 become negligible as follows:

$$-\frac{f_0}{4ka_1}\int_{\phi}^{2\pi/\omega+\phi} F_{\rm pif}(z)\cos(3\omega t+\phi_1)dt\cos(\omega_{\rm m}t+\phi_{\rm m}) \sim 0, \tag{7}$$

$$\frac{f_0}{4ka_1}\int_{\phi}^{2\pi/\omega+\phi} F_{\mathrm{pif}}(z)\sin(3\omega t+\phi_1)dt\sin(\omega_{\mathrm{m}}t+\phi_{\mathrm{m}})\sim 0. \tag{8}$$

Considering that $\phi_1=\pi/2$,[7]

$$-\frac{f_0}{4ka_1}\int_{\phi}^{2\pi/\omega+\phi} F_{\mathrm{pif}}(z)\cos(\omega t-\phi_1)dt\cos(\omega_{\mathrm{m}}t+\phi_{\mathrm{m}}) = \frac{f_0}{4ka_1}\int_{\phi}^{2\pi/\omega+\phi} F_{\mathrm{pif}}(z)\cos(\omega t+\phi_1)dt\cos(\omega_{\mathrm{m}}t+\phi_{\mathrm{m}}) \tag{9}$$

In addition, when we consider $F_{\mathrm{pif}}(z)=F_{\mathrm{pif}}(z_0)+dF_{\mathrm{pif}}/dz(z-z_0)$ as part of a small-amplitude approximation, the fourth term in Eq. (6) is transformed into

$$\begin{aligned}
&\frac{f_0}{4ka_1}\int_{\phi}^{2\pi/\omega+\phi} F_{\mathrm{pif}}(z)\sin(\omega t+\phi_1)dt\sin(\omega_{\mathrm{m}}t+\phi_{\mathrm{m}})\\
&\sim \frac{f_0}{4ka_1}\int_{\phi}^{2\pi/\omega+\phi}\{F_{\mathrm{pif}}(z_0)+\frac{dF_{\mathrm{pif}}}{dz}a_1\cos(\omega t+\phi_1)\}\sin(\omega t+\phi_1)dt\sin(\omega_{\mathrm{m}}t+\phi_{\mathrm{m}})\\
&=0+\frac{f_0}{4ka_1}\int_{\phi}^{2\pi/\omega+\phi}\frac{dF_{\mathrm{pif}}}{dz}a_1\cos(\omega t+\phi_1)\sin(\omega t-\phi_1)dt\sin(\omega_{\mathrm{m}}t+\phi_{\mathrm{m}})\\
&=\frac{f_0}{4ka_1}\int_{\phi}^{2\pi/\omega+\phi}\frac{a_1}{2}\frac{dF_{\mathrm{pif}}}{dz}\{\sin(2\omega t)+\sin(-2\phi_1)\}dt\sin(\omega_{\mathrm{m}}t+\phi_{\mathrm{m}})=0.
\end{aligned} \tag{10}$$

Therefore, substituting (6) to (10) into (5) yield.

$$\begin{aligned}
\Delta f=&-\frac{f_0}{2ka_1}\int_{\phi}^{2\pi/\omega+\phi}\{F_{\mathrm{nonpif}}(z)+F_{\mathrm{pif}}(z)\}\cos(\omega t+\phi_1)dt\\
&+\frac{f_0}{4ka_1}\int_{\phi}^{2\pi/\omega+\phi}F_{\mathrm{pif}}(z)\cos(\omega t+\phi_1)dt\cos(\omega_{\mathrm{m}}t+\phi_{\mathrm{m}})\\
&=\Delta f(0)+\Delta f(f_{\mathrm{m}}).
\end{aligned} \tag{11}$$

From formula (11), we can see that in the heterodyne-FM technique, $\Delta f(f_{\mathrm{m}})$ ($\Delta f(f_{\mathrm{m}})\mathrm{X}$) is only $-1/2$ of the $\Delta f$ in FM-AFM if we do not consider the difference in the forces. Therefore, the formula for calculating the photoinduced force ($F_{\mathrm{pif}}$) from $\Delta f(f_{\mathrm{m}})$ ($\Delta f(f_{\mathrm{m}})\mathrm{X}$) at an arbitrary amplitude in the heterodyne-FM technique is equivalent to the frequency shift-to-force conversion formula in FM-AFM multiplied by $-2$ as follows:

$$F_{\mathrm{pif}}(z)=-4k\int_z^{\infty}\left[(1+\frac{a_1^{1/2}}{8\sqrt{\pi(t-z)}})\Omega(t)-\frac{a_1^{3/2}}{\sqrt{t-z}}\frac{d\Omega(t)}{dt}\right]dt \tag{12}$$

Here, $\Omega(t)=\Delta f(f_{\mathrm{m}})/f_0$ or $\Delta f(f_{\mathrm{m}})\mathrm{X}/f_0$.

## Supplementary Note 7: Influence of surface photo-voltage

In this section, we examine the influence of the surface photo-voltage (SPV) on the PiFM observation. In the presence of the surface photo-voltage, the PiFM signal ($\Delta f(f_\mathrm{m})\mathrm{X}$) is theoretically expressed as the following equation.

$$\Delta f(f_\mathrm{m})\mathrm{X} = \frac{f_0}{4ka_1}\int_{\phi}^{2\pi/\omega+\phi}\frac{\partial C}{\partial z}\cos(\omega t+\phi_1)dtV_\mathrm{SPV}(V_\mathrm{DC}-V_\mathrm{CPD})\cos(\omega_\mathrm{m}t+\phi_\mathrm{m}) + \frac{f_0}{4ka_1}\int_{\phi}^{2\pi/\omega+\phi}F_\mathrm{pif}(z)\cos(\omega t+\phi_1)dt\cos(\omega_\mathrm{m}t+\phi_\mathrm{m}), \quad (13)$$

where the first and second terms in the right side represent electrostatic force and photoinduced optical gradient force resulting from the modulation with the frequency of $2\omega+\omega_\mathrm{m}$, respectively. Here, $C$ is the capacitance between the tip and sample, $V_\mathrm{SPV}$, $V_\mathrm{DC}$, and $V_\mathrm{CPD}$ are the induced surface photo-voltage on the surface of the QD, the applied voltage, and the contact potential difference between the tip and sample, respectively. Considering this equation and discussions of KPFM measurement in the previous papers[8, 9], $\Delta f(f_\mathrm{m})X$ is proportional to the applied bias voltage between the tip and sample ($V_\mathrm{DC}$) and it becomes 0 at $V_\mathrm{DC}=V_\mathrm{CPD}$ when the SPV is the dominant detected signal. On the other hand, when the dominant detected signal is the optical gradient force, the signal does not depend on the applied voltage and should show a constant value. $\Delta f(f_\mathrm{m})X-V$ spectra are plotted in Supplementary Fig. 11 using linear fitting. The spectra were measured on the nanorod and nanoellipsoid using 520 nm and 660 nm light illumination.

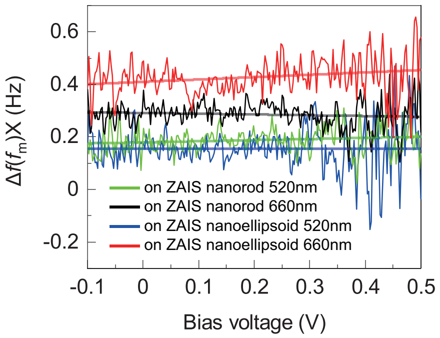


Supplementary Fig. 11: Photoinduced force signal vs. bias voltage spectra ($\Delta f(f_\mathrm{m})\mathrm{X}-\mathrm{V}$). The sites where the spectra were obtained were on the nanorod and nanoellipsoid. The wavelengths of the incident light were 520 nm and 660 nm.

All spectra show almost independent features on the applied voltage although they become unstable when the applied voltage is high. The tilts of each spectrum are 0.09006 (red), -0.02874 (black), 0.04192 (green), and 0.00224 (blue) Hz/V, respectively. Although the tilts of the spectra can be changed by the noise and the instability of the tip, they are quite small.

Then, we examine the $\Delta f-V$ spectra. Instability of the spectra in Supplementary Fig. 11 was observed in the spectra of $\Delta f-V$ as well. $\Delta f-V$ spectra measured on the nanoellipsoid of the ZAIS QD are represented in Supplementary Fig. 12.

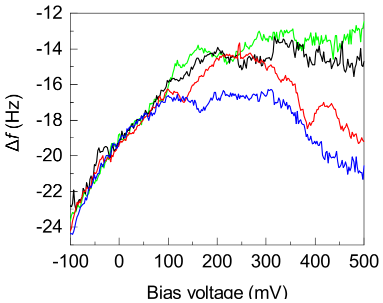


Supplementary Fig. 12: $\Delta f - \mathrm{V}$ spectra at a site of the nanoellipsoid with the illumination.

These spectra became unstable particularly when the applied voltage was high. However, by averaging these unstable spectra over many trials, we compared the $\Delta f - V$ spectra with and without the illumination using parabolic fitting as seen in Supplementary Fig.13.

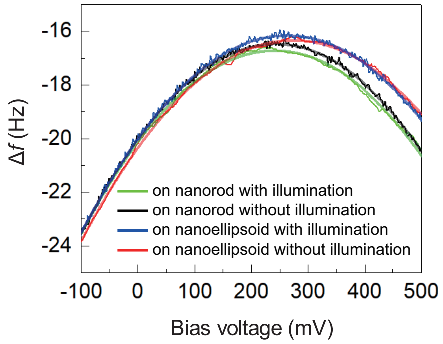


Supplementary Fig. 13: $\Delta f - \mathrm{V}$ spectra on the nanorod and nanoellipsoid with and without the illumination. The green and black plots represent the spectra on the nanoellipsoid with and without illumination, respectively. Similarly, the red and blue plots represent the spectra on the nanorod with and without illumination, respectively. The spectra with illumination were averaged over many trials.

These curves represent that the $\Delta f - V$ spectra does not show a major change with and without illumination. The respective difference of CPD with and without the illumination on the nanorod and nanoellipsoid are $\sim 10.9$ mV and $-1.25$ mV. These values are enough small to eliminate the possibility of detection of SPV.

## Supplementary Note 8: Interaction potential

The interaction potential of the photoinduced force is obtained by integrating the obtained force over the distance between the tip and the sample. Supplementary Fig. 14 shows a calculated interaction potential mapping.

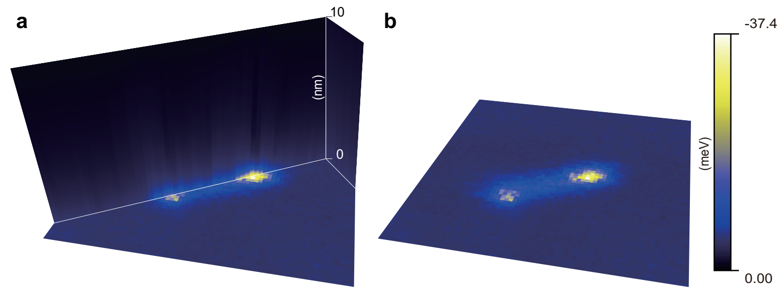


Supplementary Fig. 14: Three-dimensional mapping of the photoinduced interaction potential in the measurement with tip–sample distance feedback control ($\Delta f = -28$ Hz, $A = 10$ nm, $\lambda = 660$ nm, and $P = 15 \pm 15$ mW). **a**, $x$, $y$, $z$ space. **b**, $x$, $y$ plane ($\Delta z = 0$ nm).

## Supplementary Note 9: Lateral force

The photoinduced force in the lateral direction between the tip and the sample can be obtained by numerically differentiating the interaction potential in the $x$ and $y$ directions. In this study, this numerical differentiation was performed using the Savitzky–Golay differential filter (5 points, 1 order). Supplementary Fig. 15 shows the calculated force mapping with regard to the $x$, $y$, and $z$ directions.

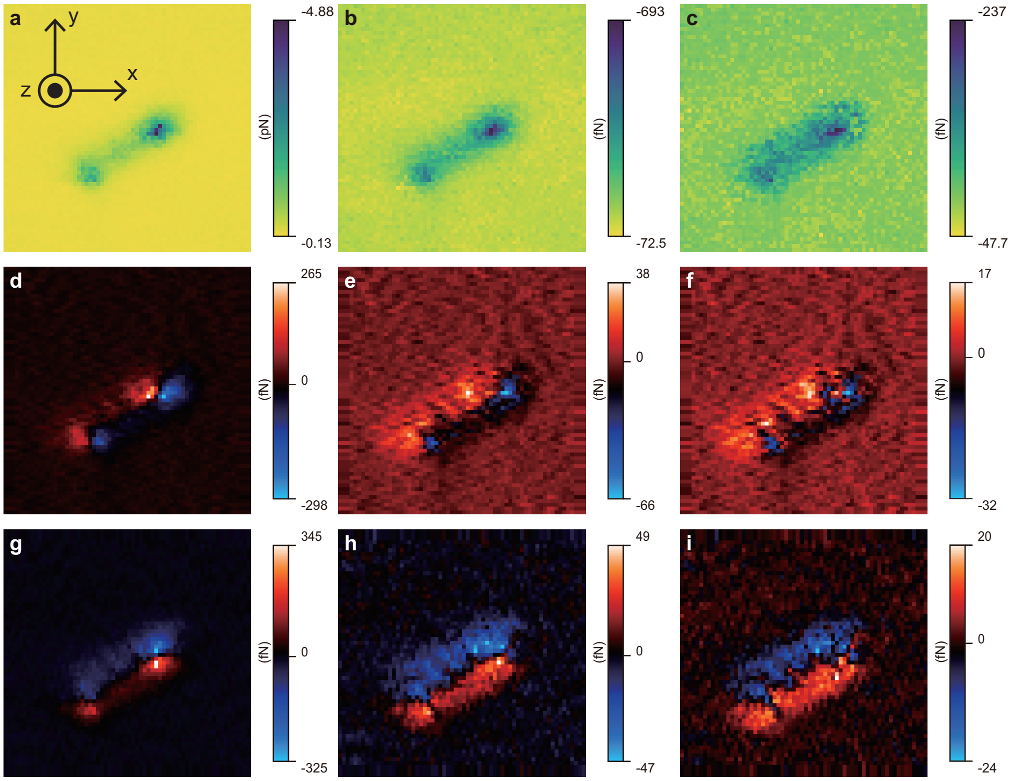


Supplementary Fig. 15: Three-dimensional force field mapping of the photoinduced force in the measurement with tip–sample distance feedback control ($\Delta f = -28$ Hz, $A = 10$ nm, $\lambda = 660$ nm, and $P = 15 \pm 15$ mW). **a**-**c**, Photoinduced force ($Fz_{\mathrm{pif}}$). **d**-**f**, Photoinduced force ($Fx_{\mathrm{pif}}$). **g**-**i**, Photoinduced force ($Fy_{\mathrm{pif}}$). In **a**, **d**, and **g**, $\Delta z = 0.0$ nm. In **b**, **e**, and **h**, $\Delta z = 1.0$ nm. In **c**, **f**, and **i**, $\Delta z = 2.0$ nm.

## Supplementary Note 10: Three dimensional photoinduced force map for different wavelength

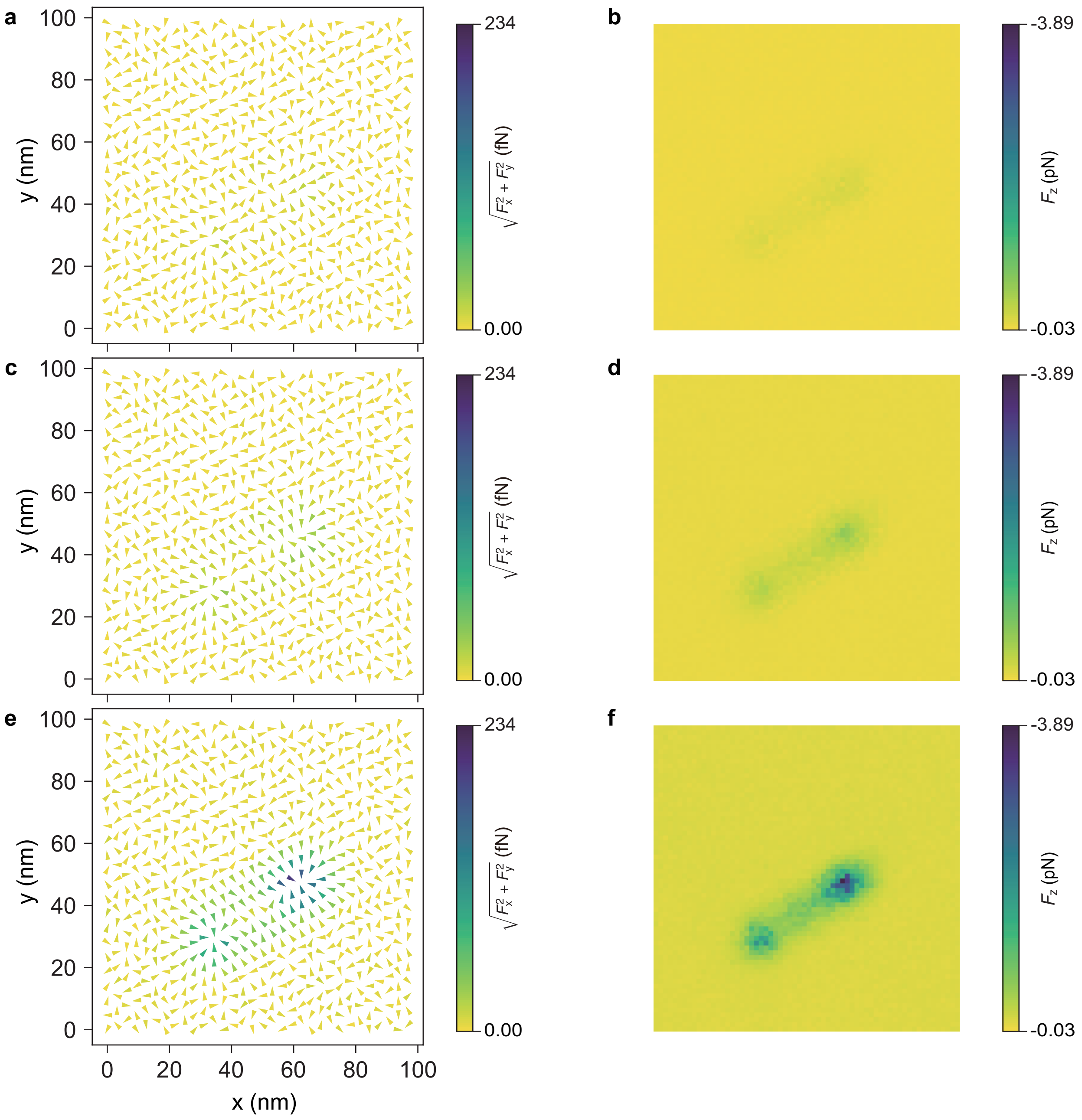


Supplementary Fig. 16: Three dimensional photoinduced force map using laser with 520 nm wavelength. **a**, **c**, **e**, Photoinduced force of $Fx, y_{\mathrm{pif}}$. **b**, **d**, **f**, Photoinduced force $Fz_{\mathrm{pif}}$. **a**, **b**), **c**, **d**), and **e**, **f**) are for $\Delta z = 2.0$, $1.0$, and $0.0$ nm, respectively.

## Supplementary Note 11: Theoretical method

In this part of the Supplementary information, we provide a theoretical treatment of photoinduced force mapping. We obtained the self-consistent total response electric field induced in a nanogap made of a metal probe, a QD and a metal substrate using discrete dipole approximation (DDA)[10] as the calculation method. The following equations were used for the calculation:

$$\boldsymbol{E}(\boldsymbol{r}_i, \omega) = \boldsymbol{E}^0(\boldsymbol{r}_i, \omega) + \int_V d\boldsymbol{r}_j \boldsymbol{G}(\boldsymbol{r}_i, \boldsymbol{r}_j, \omega)\boldsymbol{P}(\boldsymbol{r}_j, \omega), \tag{14}$$

$$\boldsymbol{P}(\boldsymbol{r}_i, \omega) = \chi_i(\omega)\boldsymbol{E}(\boldsymbol{r}_i, \omega), \tag{15}$$

where $\boldsymbol{E}(\boldsymbol{r}_i, \omega)$ and $\boldsymbol{E}^0(\boldsymbol{r}_i, \omega)$ represent the total response field and the incident field, respectively, $i$ is the number of the cell at coordinate $\boldsymbol{r}_i$, and $\omega$ is the angular frequency of the electric field. $\boldsymbol{G}(\boldsymbol{r}_i, \boldsymbol{r}_j, \omega)$ is the free space Green function with both transverse and longitudinal electromagnetic components. $\boldsymbol{P}(\boldsymbol{r}_j, \omega)$ is the polarisation density of the $j^{\text{th}}$ cell. The integral of the second term in Eq. (14) represents the field at the $i^{\text{th}}$ cell propagated from the polarisation at the $j^{\text{th}}$ cells, and $V$ is the volume of one cell. $\chi_i(\omega)$ is the optical susceptibility.

We assumed that the gold-coated tip is a gold sphere with a diameter of 19 nm and that the gold substrate is a gold thin film of $143 \times 143 \times 9\ \mathrm{nm}^3$ in this numerical calculation. The metallic structures were assumed to have a Drude-type susceptibility with the parameters of Au,[11]

$$\chi^{\mathrm{Au}}(\omega) = \frac{1}{4\pi}\left[\epsilon_{\mathrm{B}} - \epsilon_0 - \frac{(\hbar\omega_{\mathrm{Au}})^2}{(\hbar\omega)^2 + i\hbar\omega\left(\hbar\gamma_{\mathrm{bulk}} + \frac{\hbar V_{\mathrm{F}}}{L_{\mathrm{eff}}}\right)}\right], \tag{16}$$

where $\epsilon_{\mathrm{B}}$ is the background dielectric constant of the metal and $\epsilon_0$ is the dielectric constant of vacuum. $\omega_{\mathrm{Au}}$ is the bulk plasma frequency, and $\gamma_{\mathrm{bulk}}$ is the electron relaxation constant of the bulk gold. $V_{\mathrm{F}}$ is the electron velocity at the Fermi level. $L_{\mathrm{eff}}$ is the effective mean free path of electrons, comparable to the size of the tip diameter (we assumed that $L_{\mathrm{eff}} = 20\ \mathrm{nm}$). We used the parameters $\epsilon_{\mathrm{B}} = 12.0$, $\epsilon_0 = 1.0$, $\hbar\omega_{\mathrm{Au}} = 8.958$ eV, $\hbar\gamma_{\mathrm{bulk}} = 72.3$ meV, and $\hbar V_{\mathrm{F}} = 0.922\ \mathrm{nm} \cdot \mathrm{eV}$.

For the ZAIS QD, we assumed that the nanoellipsoids of the QD are ellipsoid spheres with the major and minor axes of 9 and 5 nm, respectively, while the nanorod of the QD is a cylinder with the major and minor axes of 23 and 3 nm, respectively. The dielectric function of the QD was determined from the molar absorption spectrum of the solution obtained by experimental measurement using the Kramers-Kronig relation. In the measurement, the spectrum could not be obtained at 240 nm or lower because of the absorption of the solvent. Therefore, we extrapolated the absorption spectrum at 150 to 240 nm by approximating the short wavelength region (240 to 350 nm) of the measurement spectrum with a power function.

## Supplementary Note 12: Theoretical analysis of resolution

Here, we perform calculations to show the resolution of the experimental results. Using an extended method of discrete dipole approximation developed by the present authors,[12] we simulate the photoinduced force reflecting the geometrical structure of the tip apex. In this method, we can model the sample structure and evaluate the spatial structure of the electric field in the angstrom scale for the part of the simulation volume we are focusing on via the multiple-cell-size method. (In the main text, we did not use this method to avoid a heavy cumulative computational load.)

We calculate the photoinduced force gradient ($\Delta F z_{\rm pif}/\Delta z$), which is approximately proportional to the amount measured via PiFM ($\Delta f(f_{\rm m})$X),[7] when the distance between the tip and the sample is $0.4 \sim 0.5$ nm, assuming that the ZAIS rod has protrusions with a diameter of approximately 1 nm. At this distance, the wave functions of the tip and sample do not overlap; therefore, the classic model of a metal tip can be used safely .[13] We compare the two tip models; one is a tip model of a sphere with a diameter of about 60 nm, part of the volume of which is divided into cubic cells with a length of 1 Å, and the other is a tip with a sphere that has a nanoscale protrusions as shown in Supplementary Fig. 17a and b, respectively. In the former case, the tip of the probe is flat. As is well known, the tip of a gold-coated probe has many nanoscale protrusions; this assumption is reasonable regarding the latter case rather than the former case. It is known that individual nanoscale features inside the metal nanogap, such as protrusions on the tip, can induce highly localised and enhanced electric fields within volumes well below 1 $\rm nm^3$, thereby enabling optical experiments on the nanoscale.[14] Such atomic structures are also called "picocavities." In Supplementary Fig. 17c and d, we show the line profiles of the force gradient with regard to the tips with and without protrusions. We can conclude that our theoretical examination successfully verifies the resolution of our method. Thus, we are convinced that our experiment achieved a resolution of approximately 0.7 nm.

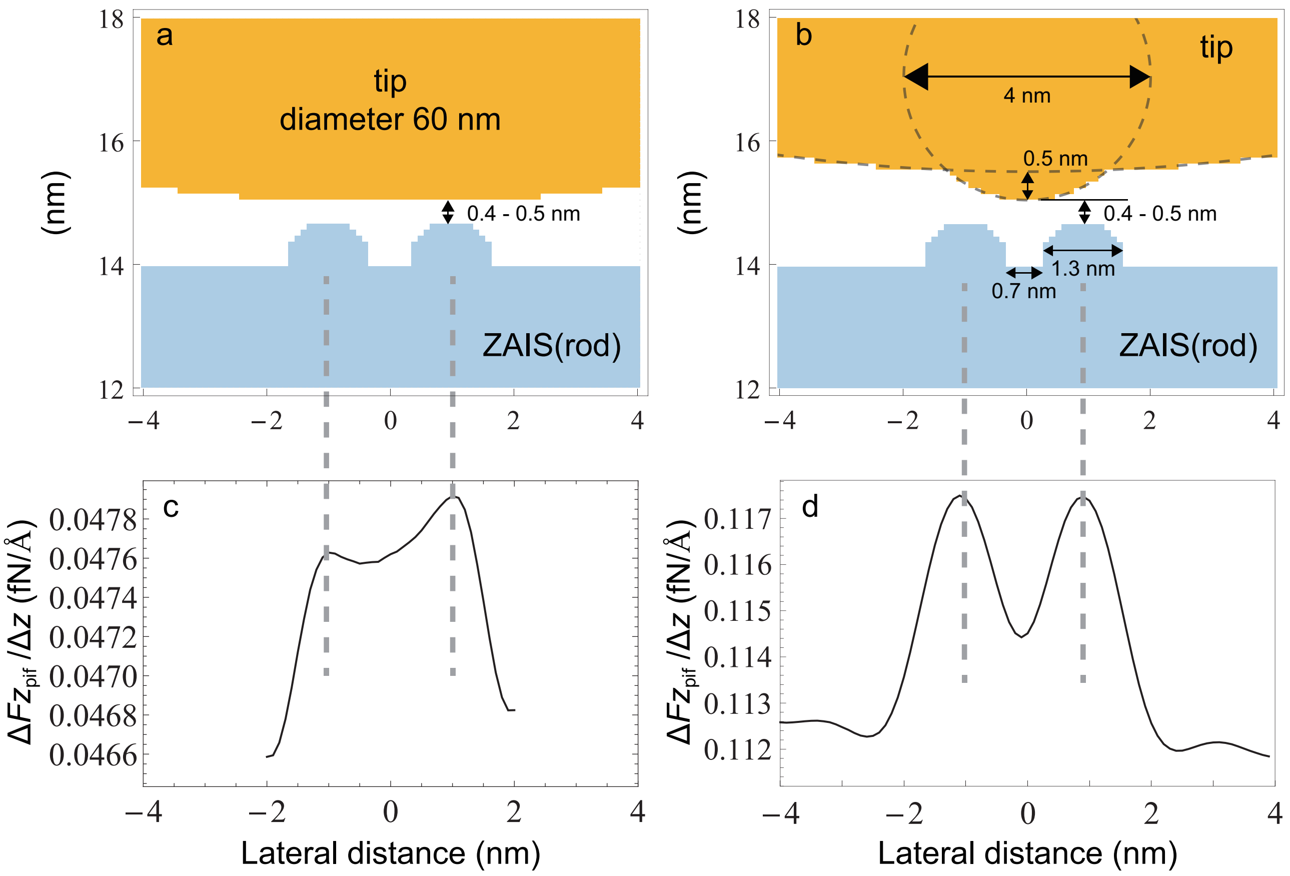


Supplementary Fig. 17: **a**-**b**, schematic models of the tips with and without protrusions (picocavities), respectively. The orange and the light blue areas of **a** and **b** represent the tip and the rod part of ZAIS, respectively. **c**-**d**, the line profiles of the gradient of the photoinduced force for **a** and **b**.

## Supplementary Note 13: Magnitude of photoinduced force

The magnitude of the photoinduced force, obtained via the experimental result, is 3500 times larger in the horizontal direction and 9600 times larger in the vertical direction than that obtained via the theoretical calculation. In the present study, the fitting of the absolute value is not essential because there are several factors related to the tip that do not affect the essence of the present discussion; in addition, the light intensity near the sample is uncertain. (The force is simply proportional to the light intensity.) Nevertheless, we can provide a plausible discussion in this regard, as follows: We have presented the results of the photoinduced force calculated by assuming that the tip has a diameter of 19 nm in the main text because of the calculation cost. In contrast, for a tip with diameter of 60 nm, which is the size that was used in the experimental measurements, the photoinduced force is approximately 60 times larger than the theoretically calculated force. Supplementary Fig. 18 shows the tip-size dependence of the photoinduced force acting on the nanorod.

Additionally, in the experiment, the distance between the tip and the QDs approaches 0 nm, whereas in the calculation, the tip is separated by 1 nm from the QDs. If the tip–QD distance approaches 0 nm, then the force becomes approximately 10 times that at 1 nm (see Fig. 3b in the main text). Multiplying these factors increases the force by approximately 600 times. Another factor is the shape of the tip. In our calculation, the tip was assumed to be spherical. If we consider the length of the tip, the force becomes approximately 3 times larger according to our calculation. If we also consider the uncertainty of the light intensity at the apex of the tip, the present discrepancy in the induced force obtained via the calculation and the observed results will be resolved (although the laser intensity of $10\ \mathrm{kW/cm^2}$ that we have used in the calculation is reasonable). Hence, we consider that comparing the absolute strength of the photoinduced force obtained via the experiment and calculation in this study does not prove to be beneficial in the context of this study.

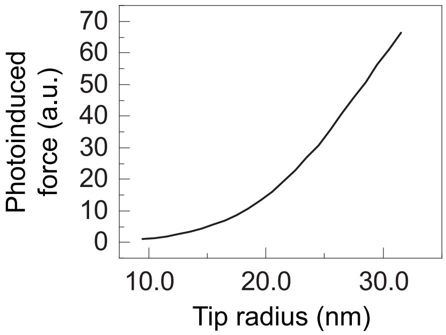


Supplementary Fig. 18: Tip-size dependence of the photoinduced force on the nanorod. The vertical axis represents the ratio of the photoinduced force and the force acting on the tip with a 19 nm diameter.

## Supplementary Note 14: Theoretical calculation of the photoinduced force vectors

The theoretical calculation of the 3D-mapped photoinduced force field vectors is performed as follows. In this calculation method, we do not evaluate the forces at tip–sample distance $\Delta z = 0$ nm because the DDA mesh size is set to $1\ \mathrm{nm}^3$. Thus, the forces at a distance of $\Delta z = 1$ nm or more are shown in Supplementary Fig. 19. When comparing this with the experimental result of Fig. 4a-f, it should be noted that the maximum values of the vector lengths in Fig. 4a are based on the force at $\Delta z = 0$ nm. In agreement with the experimental data in

the main text, the photoinduced force vectors are directed toward two spots, i.e., the edges of the ZAIS QDs.

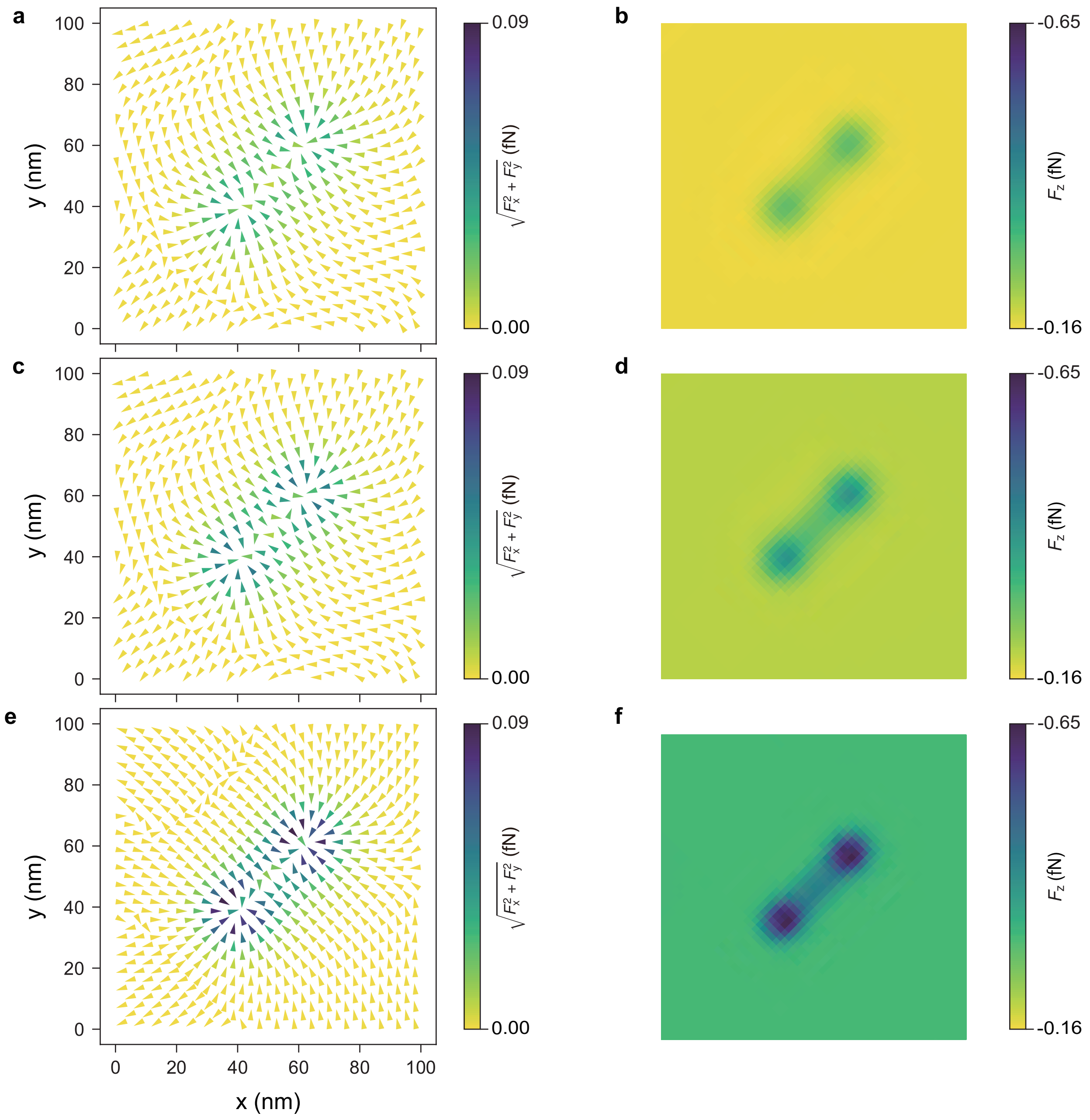


Supplementary Fig. 19: Theoretical calculation of the three dimensional photoinduced force field vectors. **a**, **c**, **e**, Photoinduced force map directing $x$ and $y$ at $\Delta z = 3, 2, 1$ nm. The directions and colours of the arrows represent their directions and magnitudes. **b**, **d**, **f**, Photoinduced force map directing $z$ at $\Delta z = 3, 2, 1$ nm. The wavelength is 660 nm.

## Supplementary Note 15: Gradient of electric field intensity in the absence of the tip

The theoretically calculated map of the intensity gradient of the electric field polarised along the $z$-axis ($\nabla|\boldsymbol{E}z|^2$) in the absence of the tip is shown in Supplementary Fig. 20. The arrows shown in the Supplementary Fig. 20a represent $\nabla|\boldsymbol{E}z|^2$ in the $x$ and $y$ directions. Supplementary Fig. 20b represents $\nabla|\boldsymbol{E}z|^2$ in the $z$ direction. The colour of each arrow indicates the strength of the corresponding $x$-$y$ $\nabla|\boldsymbol{E}z|^2$ vector. The sharper variations of the field-intensity gradient around the ZAIS QD than those of the force field vectors in Fig. 4 in the main text are due to the absence of the tip in this calculation.

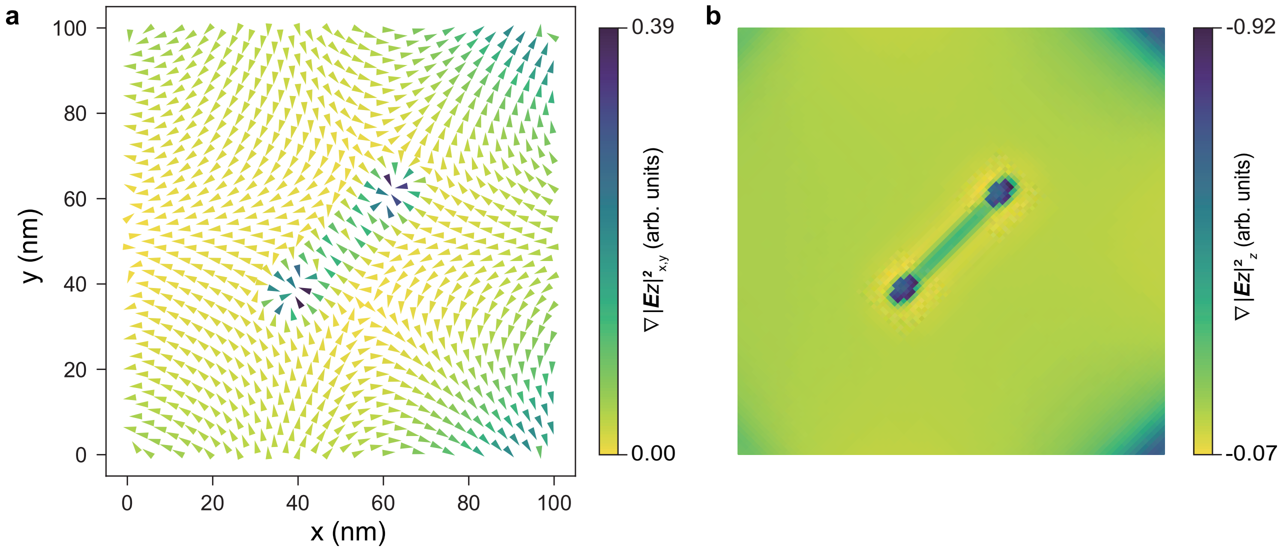


Supplementary Fig. 20: Theoretically calculated map of the intensity gradient of the electric field polarized along the $z$-axis in the absence of the tip. **a**, the map of the gradient directing $x$ and $y$. **b**, the map of the gradient directing $z$. The wavelength is 660 nm.

## Supplementary References


[1] Brian T O'Callahan, Jun Yan, Fabian Menges, Eric A Muller, and Markus B Raschke. Photoinduced tip–sample forces for chemical nanoimaging and spectroscopy. *Nano letters*, 18(9):5499–5505, 2018.

[2] Junghoon Jahng, Eric O Potma, and Eun Seong Lee. Tip-enhanced thermal expansion force for nanoscale chemical imaging and spectroscopy in photoinduced force microscopy. *Analytical chemistry*, 90(18): 11054–11061, 2018.

[3] Lukas Novotny and Bert Hecht. *Principles of nano-optics*. Cambridge university press, 2012.

[4] Junghoon Jahng, Jordan Brocious, Dmitry A Fishman, Fei Huang, Xiaowei Li, Venkata Ananth Tamma, H Kumar Wickramasinghe, and Eric Olaf Potma. Gradient and scattering forces in photoinduced force microscopy. *Physical Review B*, 90(15):155417, 2014.

[5] Feng Lu, Mingzhou Jin, and Mikhail A Belkin. Tip-enhanced infrared nanospectroscopy via molecular expansion force detection. *Nature photonics*, 8(4):307, 2014.

[6] Vasily Kravtsov, Samuel Berweger, Joanna M Atkin, and Markus B Raschke. Control of plasmon emission and dynamics at the transition from classical to quantum coupling. *Nano letters*, 14(9):5270–5275, 2014.

[7] Seizo Morita, Franz J Giessibl, Ernst Meyer, and Roland Wiesendanger. *Noncontact atomic force microscopy*, volume 3. Springer, 2015.

[8] Osamu Takeuchi, Yoshihisa Ohrai, Shoji Yoshida, and Hidemi Shigekawa. Kelvin probe force microscopy without bias-voltage feedback. *Japanese Journal of Applied Physics*, 46(8S):5626, 2007.

[9] Ch Sommerhalter, Th Glatzel, Th W Matthes, A Jäger-Waldau, and M Ch Lux-Steiner. Kelvin probe force microscopy in ultra high vacuum using amplitude modulation detection of the electrostatic forces. *Applied surface science*, 157(4):263–268, 2000.

[10] E. M. Purcell and C. R. Pennypacker. *Astrophys. J.*, 186:705, 1973.

[11] P. B. Johnson and R. W. Christy. *Phys. Rev. B*, 6:4370, 1972.

[12] Mai Takase, Hiroshi Ajiki, Yoshihiko Mizumoto, Keiichiro Komeda, Masanobu Nara, Hideki Nabika, Satoshi Yasuda, Hajime Ishihara, and Kei Murakoshi. Selection-rule breakdown in plasmon-induced electronic excitation of an isolated single-walled carbon nanotube. *Nature Photonics*, 7(7):550, 2013.

[13] Rubén Esteban, Asier Zugarramurdi, Pu Zhang, Peter Nordlander, Francisco J García-Vidal, Andrei G Borisov, and Javier Aizpurua. A classical treatment of optical tunneling in plasmonic gaps: extending the quantum corrected model to practical situations. *Faraday discussions*, 178:151–183, 2015.

[14] Felix Benz, Mikolaj K Schmidt, Alexander Dreismann, Rohit Chikkaraddy, Yao Zhang, Angela Demetriadou, Cloudy Carnegie, Hamid Ohadi, Bart De Nijs, Ruben Esteban, et al. Single-molecule optomechanics in “picocavities”. *Science*, 354(6313):726–729, 2016.
